# Hepatic Metastasis from Colorectal Cancer

**DOI:** 10.5005/jp-journals-10018-1241

**Published:** 2017-09-29

**Authors:** Alan I Valderrama-Treviño, Baltazar Barrera-Mera, Jesús C Ceballos-Villalva, Eduardo E Montalvo-Javé

**Affiliations:** 1 Department of Surgery, Universidad Nacional Autonoma de México, Ciudad de México, México; 2 Department of Physiology, Universidad Nacional Autonoma de México, Ciudad de México, México; 3 AFINES, Faculty of Medicine, Universidad Nacional Autonoma de México Ciudad de México, México; 4 Department of General Surgery, Hospital General de México, Ciudad de México, México

**Keywords:** Colorectal cancer, Hepatic metastasis, Treatment of metastasis.

## Abstract

The liver is the most common site of metastasis in patients with colorectal cancer due to its anatomical situation regarding its portal circulation. About 14 to 18% of patients with colorectal cancer present metastasis at the first medical consultation, and 10 to 25% at the time of the resection of the primary colorectal cancer. The incidence is higher (35%) when a computed tomography (CT) scan is used.

In the last decades, a significant increase in the life expectancy of patients with colorectal cancer has been achieved with different diagnostic and treatment programs. Despite these improvements, the presence of metastasis, disease recurrence, and advanced local tumors continue to remain poor prognostic factors.

Median survival without treatment is <8 months from the moment of its presentation, and a survival rate at 5 years of 11% is the best prognosis for those who present with local metastasis. Even in patients with limited metastatic disease, 5-year survival is exceptional. Patients with hepatic metastasis of colorectal cancer have a median survival of 5 to 20 months with no treatment. Approximately 20 to 30% of patients with colorectal metastasis have disease confined to the liver, and this can be managed with surgery. Modern surgical strategies at the main hepatobiliary centers have proved that hepatectomy of 70% of the liver can be performed, with a mortality rate of <5%.

It is very important to have knowledge of predisposing factors, diagnostic methods, and treatment of hepatic metastasis. However, the establishment of newer, efficient, preventive screening programs for early diagnosis and adequate treatment is vital.

**How to cite this article:** Valderrama-Treviño AI, Barrera-Mera B, Ceballos-Villalva JC, Montalvo-Javé EE. Hepatic Metastasis from Colorectal Cancer. Euroasian J Hepato-Gastroenterol 2017;7(2):166-175.

## INTRODUCTION

Colorectal cancer is the third most common cancer in the world in terms of incidence and the fourth in mortality,^1^ immediately behind lung, liver, and stomach cancer.^2^ It occupies the second most common type of cancer in women and the third in men, representing 9.7% worldwide of all types of cancer excluding nonmelanomatous skin cancer. In 2012, 614,000 new cases were reported in women and 746,000 in men. Geographically, it is more common in developed countries, with Australia and New Zealand being the countries with highest incidence (44.8 and 32.2 for 100,000 habitants respectively). Meanwhile, in the US, there are 145,000 new cases annually,^3^ of which 11% are newly diagnosed. In the UK,^4^ 10% of deaths are related to cancer, with a mean average age of 50 years in 75% of cases.^5^

Mortality rate by age in colorectal cancer is higher in men than in women, and is also twofold in developed (11.6/100,000 habitants) in comparison with developing countries (6.6/100,000 inhabitants), highlighting the region of Eastern Europe, with the highest mortality rate for men and women.

Worldwide mortality has increased from 1990 to 2013 (57%); however, in some regions of Europe, North America, and Asia, this has decreased, primarily due to the application of screening methods, such as colo-noscopy, which is related with early diagnosis and treatment.^5^

**Fig. 1:**
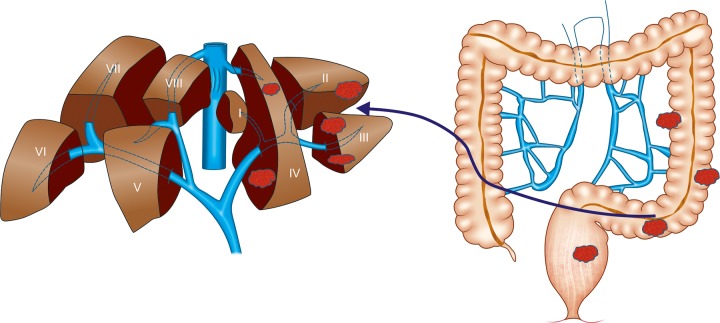
Hepatic metastases from colorectal cancer

The liver is the most common site of metastasis in patients with colorectal cancer due to its anatomical situation with regard to portal circulation^3^ (Fig. 1). About 14 to 18% of the patients with colorectal cancer present metastasis at the first medical consultation^6^ and 10 to 25% are diagnosed at the time of primary tumor resection. The incidence is higher (35%) when patients undergo CT scan or ultrasound (US) imaging. Eventually, about 70% of patients with colorectal cancer will develop metastasis in the liver.^7^ From among 1,450,000 patients with colorectal cancer with a recent diagnosis of colorectal cancer, it is expected that 30,000 to 40,000 will develop synchronic metastasis (one-third of cases) or metachronous metastasis (two-third of cases) confined to the liver.^3^ ^8^

In recent decades, an increase in the life expectancy of patients with colorectal cancer has been achieved, with screening, early diagnosis, novel chemotherapy agents, and improvements in radiotherapy and in surgical techniques. Despite these improvements, poor prognostic factors continue to include the presence of metastasis, disease recurrence, and advanced localized tumors,^9^ reporting that one-half of these patients with these risk factors die in the presence of these neoplasms.^10^ Colorectal cancer is one of the most common diseases, with approximately one million new cases and 500,000 deaths annually worldwide, the main cause of death being metastasis. Mortality rates vary depending on the stage at which the neoplasm is diagnosed, this representing a public health issue.^5^

Due to its location, size, the amount of metastases in the liver, normal residual liver, and the additional hepatic disease, 85% of these patients are not eligible for surgery.^10^

## RISK FACTORS

Genetic and epigenetic factors have great relevance for the disease. The majority of cancers are sporadic: Approximately 75% of patients have a negative history. In Western populations, a risk has been reported to be of 3 to 5%; this risk is twofold in persons with a first-degree familial history diagnosis at between 50 and 70 years of age, and threefold in those whose relative was diagnosed prior to the age of 50 years. Positive familial history is present in 15 to 20% of patients with colorectal cancer, this being the hereditary syndrome of colorectal cancer in 5 to 10% of patients. Lynch syndrome is the most common in this category, which is caused by a mutation in one of the following deoxyribonucleic acid (DNA) repair genes: MLH1; MSH2; MSH6; PMS2, or EPCAM. The defects in these genes give rise to a lack of the main repair mechanisms during replication, and in consequence, there is an accumulation of mutations in the genetic material. This takes places predominantly in microsatellite fragments of DNA, rendering diagnosis possible with polymerase chain reaction (PCR) tests comparing normal DNA against the carrier of microsatellite instability. Familial adenomatous polyposis represents the second most common cause of familial syndromes, caused by a mutation in the APC gene that controls activity in the WNT signal pathway. This syndrome is characterized by the development of colorectal adenomas and the presence of colorectal cancer at an early age. Other causes include polyposis related with mutations of the MUTY DNA glucosylase (MUTYH), the Peutz-Jeghers syndrome, sawtooth polyposis, and juvenile polyposis. Chronic colitis is another cause to consider, in that it is associated with 1% of cases of colorectal cancer in Western population. In several studies, it was reported that the incidence of colorectal cancer can be diminished with proper antiinflammatory treatment; this also shows an improvement in survival rate.

The risk of colorectal cancer increases with tobacco consumption (20-50%), alcohol consumption (20-50%), overweight and obesity (2-3% for every unit in body mass index), diabetes mellitus, and the consumption of red meat and processed foods (1.16 times for each 100 gm increase in the daily diet). It is estimated that lifestyle is related with between 16 and 71% of cases of colorectal cancer in Europe and the US. The use of statins could exert some preventive effect, as well as the use of hormone replacement therapy for postmenopausal women.^5^ The different environmental factors that influence carcinogenesis is reflected in the heterogeneity of colorectal cancer and it stimulates the investigation field of molecular pathological epidemiology.

## NATURAL HISTORY OF THE DISEASE

Colorectal cancer is one of the most common types of cancer in Western populations. The liver is the first location of metastatic disease; due to that the main mechanism of dissemination is through the portal system. In addition, the liver may be the sole site of metastasis in 30 to 40% of patients with advanced disease.^4,11^

Unfortunately, 20% of these patients will develop metastasis in the lungs and >50% in liver.^12^ In 20 to 25% of patients at the time of diagnosis, hepatic metastatic disease can be identified clinically, and 40 to 50% will develop during the first 3 years after the primary tumor is diagnosed.^13-16^ Pulmonary or liver metachronous metastasis will develop in 8 to 13% of patients after surgical resection.^17^ Median survival without treatment is <8 months after disease presentation, and with a 5-year survival rate of 11% or less. Patients who present with isolated metastasis or metastasis confined to one hepatic lobe have a better prognosis. However, in the reports of patients with limited metastatic disease, 5-year survival is exceptional.^4^ Patients who present with metastasis to the liver have a median survival of 5 to 20 months without treatment, with 2-year survival unusual and 5-year survival extremely rare. Hepatic metastatic disease has been reported in 56% of patients with colorectal cancer in the autopsy, and in 35% of patients with isolated hepatic metastasis.^18^

Wood et al^19^ reported, in a retrospective study, that 113 patients who presented extended hepatic disease had a survival rate of 5.7%: 27% for those with metastasis in one hepatic lobe, and 60% in those with isolated metastasis. Peritoneal metastases are present in 20% of colorectal cancers and the former represents 40 to 70% of all recurrent disease. About 10 to 30% of recurrent disease is limited to peritoneum without distant metas-tasis.^20^ Patients with four or more metastases have worst prognosis, as well as those presenting large metastases, specific localization, differentiation, and lymph-node involvement.^21^ Metastatic lesions regularly respect the hepatic capsule and intersegmental layers, thus respecting nearby structures.^12^

When metastatic lesions are localized in the liver, which corresponds to 30% of patients, there are several options for localized treatment, such as hepatic partial resection, localized ablative therapy, administration of chemotherapy by infusion of the hepatic artery, systemic chemotherapy, and isolated hepatic fusion for patients with high doses of chemotherapy.^7^ Surgical resection is the most effective treatment for hepatic metastasis in colorectal cancer, but only a few patients are candidates for initial surgery.^1^ Patients with hepatic metastasis that cannot be surgically resected are managed initially with chemotherapy and later are subject to surgery, and these patients present a similar survival rate to those undergoing surgery initially.^22^ Prior to hepatic resection, patients with hepatic metastatic disease frequently receive neoadjuvant chemotherapy, which can aid in "disappearing" or hidden radiological lesions. Several studies have proven a persistent viable tumor at the site where the "lesions disappear."^18^ Advances in systemic chemotherapy and molecular therapy have achieved a prolonged survival rate, resulting in the indication of hepatic resection with neoadjuvant therapy and conversion therapy.^17^ Strategies have been implemented to increase the number of patients who are considered for complete surgical resection, such as neoadjuvant chemotherapy, preoperative embolization of the portal vein, and two-phase resection. However, even with the use of these techniques, the majority of patients with hepatic metastasis of colorectal cancer are not candidates for a curative resection.^12^

At present, hepatic resection is the only surgical technique, i.e., proven to cure hepatic metastasis.^21^ Surgical treatment of isolated metastasis is a very well-established treatment for selected patients, and it achieves a 5-year survival in between 39 and 58% of patients.^13,14^ Approximately 20 to 30% of patients with metastatic colorectal cancer have this disease isolated to the liver, and it can be surgically managed. It has been reported that 5-year survival rate after hepatic-metastasis surgical procedure is 25 to 44%, with an intraoperative mortality of 0 to 6.6%.^4^ Modern surgical techniques performed at main hepatobi-liary centers have demonstrated that a 70% hepatectomy can be achieved with a mortality rate of <5%.^23,24^

The traditional surgical strategy for synchronic hepatic metastasis of colorectal cancer is approached in two phases that include resection of the colorectal cancer followed by chemotherapy, and a delayed hepatic resection. This approach could result in the progression of hepatic disease from the time of the colorectal resection until the hepatectomy is performed. Even when the surgical resection is performed with curative intention, 60 to 70% of cases will develop local or distant recurrence.^12^ An association has been described with steatosis and a postoperative increase of morbidity and mortality, this occurring mainly in large resections.^25^

Cure is considered after a 10-year survival without the disease, and recurrent disease at this point is less likely.^26^ With the aid of hepatic resections, the 5-year survival rate, even in those with a positive margin or of <1 mm, could be increased up to 25%. In cases in which sufficient can be obtained (<1 mm), 5-year survival rates could be up to 40%. However, sometimes hepatic resections cannot be performed, due to the high number of hepatic metastases or to tumor invasion into the main vascular structures or neighboring organs.^9^

Rupertus et al^27^ demonstrated, in a standardized model, that the growth of extrahepatic tumors is correlated with the extension of the hepatic resection, due to improvements in neovascularization and tumor-cell migration. Experimental trials have demonstrated greater growth in hepatic metastases after a small (30%) and major (60-70%) hepatotectomy.^24^

In several reports, the authors studied the impact of the volume resected associated with the survival of patients with colorectal-cancer hepatic metastasis, concluding that the patient survival is negatively correlated with the extension of the hepatic resection.^24^ In a prospective study, it was proven that simultaneous resection of the liver and colon is safe and effective. By avoiding a second laparotomy, the global rate of complications is lower and the treatment timeline is shortened.^3^ Approximately 30% of patients achieved survival after hepatic resection.^21^ About 80% of the relapsing disease appears to occur during the first 2 years after the treatment, eventually developing secondary metachronous metastasis in 50% of patients managed with primary resection.^12^

Even after curative resection, 30 to 40% of patients will develop distant metastasis depending on the stage of the primary tumor.^23^ The compromise of the liver’s lymphatic nodules of the liver after the hepatic-metastasis curative resection is known to confer a dismal prognosis.^28^ Repeating the resection is feasible in 10 to 15% of cases and can reach a global 5-year survival of 15 to 40% in selected patients, despite that a median survival of patients with recurrent disease of 8 to 10 months without treatment has been documented.^12^ Chemotherapy administered systemically or locally plays a palliative role and is rarely significant for prolonged survival. Even with the improvement in the chemotherapy and biological agents, survival is rarely >3 years.^29^

## MOLECULAR ASPECTS

The metastatic expansion of the tumor cells is one of the most common causes of mortality in patients with cancer. Elucidation of the molecular mechanisms that participated in the formation of colonic metastasis has comprised one of the main objectives of cancer research. Colorectal cancer is one of the most common causes of mortality related with cancer. However, the regulatory processes of the metastasis of colorectal cancer are yet to be determined. KAI1/CD82, a member of Transmem-brane superfamily 4 (Tetraspanin), has been associated with the formation of hepatic metastasis of malignant melanoma. Transfection of colorectal-cancer CT-26 cells with this variant produces reorganization of the cytoskel-eton, resulting in earlier hepatic metastasis. The tyrosine-phosphatase PRL-3 is considered a significant marker for hepatic metastasis. The increase in PRL-3 expression was found in hepatic metastatic colorectal cancer, with the downregulation in colon cancer DLD-1, stopping metastasis to the liver without affecting cellular proliferation, while its transfection increased metachronous hepatic metastasis.^30^ Teramae et al^31^ investigated insulin-like growth factors (IGFs) I and II colorectal cancer. Blockage of IGF I/II employing antibodies has diminished the formation of hepatic metastases. The PCR studies have been performed, which have confirmed that PRDX4, CSKS2, MAGED2, and GenBank BF696304 expressed sequence tag expressed at high levels with metastatic tumors. This datum should help understand the progression of colorectal cancer and facilitates the prediction of their potential metastasis.^32^

Metastasis of the liver from colorectal cancer represents the final stage of a multistep biological process. This process begins with a series of mutations in the epithelial cells of the colon, continues with detachment of the cells, diffusion through blood or lymphatic circulation, attachment to hepatic sinusoids, and interaction with sinusoid cells, such as Kupffer cells, stellate cells, and cells in well. The metastatic sequence terminates with the invasion of colorectal cancer cells and adaptation and colonization of the hepatic parenchyma.^33^ Downregulated genes, such as ADAMTS9 and COL6A1 have been found in hepatic metastasis. COL6A1 belongs to a collagen family, and it has been reported to be associated with metastasis of meduloblastoma, breast, and prostatic cancer; however, it has also been found in hepatic metastasis. Another highly expressed gene in hepatic metastasis is *PIAS2,* a protein inhibitor of the activated STAT2 that causes a stop in the cell cycle and acts as a transcription factor that controls DNA-associated damage through several cellular pathways, such as STAT, Myc, and TP53.^34^ Understanding the mechanisms by which metastases are developed in the liver possesses great clinical significance. This can furnish useful information on the development of target drugs and individualized therapy for patients with colorectal cancer. Neoadjuvant therapies focused on the liver and the detection of a specific organ can be considered in patients labeled as high risk for the development of recurrence in liver.

## DIAGNOSIS

The clinical presentation of this pathology includes symptoms, such as fever, fatigue, anorexia, abdominal pain, a change in bowel movements, weight loss, and blood in stools. Patients also complain of abdominal fullness and right upper quadrant pain. Physical examination can reveal a mass in the liver, hepatomegaly, jaundice, and ascites. The predictive value of these signs in an older patient is limited and a thorough approach is justified, including imaging and molecular methods.^3,5^ The clinical variants, often combined with the classification systems, have proven useful as predictors in the results of treatment, with this particularly true in the area of the hepatic-metastasis resection.^35^

### Colonoscopy

Colonoscopy is the gold standard for the diagnosis of colorectal cancer, presenting high diagnostic accuracy. It allows for taking various samples, by which histological confirmation and the molecular panel can be performed. It is a technique that permits diagnosis and therapy, due to that it can eliminate adenomas by endoscopic polypectomy, thus reducing the incidence and mortality associated with cancer. The quality of the colonoscopic image has improved during the last 20 years since the employment of the optic fiber; therefore, the current standard combines the use of high-potency endoscopies and high-resolution screens for white light, high-definition endoscopy. Only chromoendoscopy has demonstrated to be superior to white light endoscopy in the identification of adenomas.^5^

### Magnetic Resonance

Adam et al indicate that magnetic resonance (MR) is more sensitive than CT for detection of hepatic lesions and, in cases of the administration of neoadjuvant chemotherapy, CT is a better option in cases in which the initial state of the tumor^1^ has been reported. Bischof et al^36^ reported a sensitivity for hepatic metastasis after chemotherapy of 85.7% for MR and of 69.9% for CT, suggesting MR as the preferred imaging study for preoperative evaluation of hepatic metastasis. Moreover, MR is useful in the detection of small lesions, as well as in defining the relation of injuries of the hepatic vasculature and biliary tree; however, it presents a sensitivity of 70 to 80% and possesses no advantage over the CT%. The Gadoxetate contrast agent has increased sensitivity in comparison with the available MR techniques, exhibiting higher sensitivity in small-sized metastases over the CT, especially in cases of hepatic steatosis. Gadoxetate is characterized as a contrast agent of the hepatobiliary system due to its high rate of absorption and excretion of functional hepatocytes, permitting adequate visibility of the lesions in the hepatobiliary phase, this being the reason for its higher sensitivity for detection of lesions in comparison with other contrast agents.^18^

Magnetic resonance should be utilized only in those who cannot tolerate the CT contrast load or for those presenting with an unspecific tomography image.^3^

### Computed Tomography

Performing a CT of the abdomen with contrast presents a detection rate of hepatic metastasis of 68 to 91%, with 70% for lesions >1 cm. Thus, in some studies, echography has been suggested as the preferred imaging technique.^1,4^ Employment of multidetector-row helical CT exhibits better resolution, with a sensitivity of 70 to 90% for detecting hepatic metastases corresponding to hypodense type in portal phase. Tomography can supply information on the anatomical characteristics of metastatic lesions and the relation of lobular architecture and vascular structures; however, it cannot detect subcentimeter lesions.^12^ Triphasic CT has demonstrated a sensitivity of >90% in the detection of the hepatic lesions in comparison with the 75% sensitivity of CT with contrast.^1^

On completion of the study of the colon with colo-noscopy, CT pneumocolon, barium enema, should be carried out during the preoperative period in patients with colorectal cancer; due to that there is a significant risk for recurrence of the lesion or a metachronous lesion.^4^ Colonography through CT has shown 96% sensitivity for detecting colorectal cancer.^1^ Plumb et al^37^ reported, in their observational study in England with 2,731 patients with a positive Guaiac test, a high detection rate of advanced neoplasms, which was significantly less for CT colonography than for conventional colonoscopy. The CT colonography requires full bowel preparation, insufflation, and change of position of the patient during the study, and entertains low sensitivity for small (6-9 mm) and flat lesions. The costs and the need for more investigation limit the use of this method as a screening tool in the majority of the population worldwide; only in the US and Europe it is employed with this purpose. It has not been fully accepted in Europe, due to exposure to radiation, costs, and the colonoscopy’s high derivative rate (30%).^1^

### Positron Emission Tomography

Positron emission tomography (PET) is one of the new technologies applied in the field of Oncology. It utilizes 18-fluoride deoxyglucose as a tracer. Selzner et al^38^ reported 88% sensitivity and 96% specificity for the detection of hepatic metastasis, while in the extrahepatic disease, this is 90 and 95% respectively. Moreover, PET is very useful in patients with recurrent disease, high tumor load (multinodular or big metastasis), or in cases in which a difficult hepatic resection is planned.^1^ Fernandez et al^39^ used PET prior to hepatic resection for metastases deriving from colorectal cancer. These authors reported a 5-year global survival of 58.6%, which is higher, to our knowledge, than that of any other study not employing PET routinely; this indicating that PET has the advantage of selecting patients who can benefit from major surgery. The combination of CT and PET increases sensitivity and enables the choice of candidates for surgical therapy who can obtain better results.^12^

The main limitation of PET is it possesses reduced sensitivity in the detection of subcentimeter lesions, mucinous lesions, and lesions previously treated with neoadjuvant chemotherapy.^4,12^

### Echography and Diagnostic Laparoscopy

Echography is a low-cost test utilized as first line in the diagnostic evaluation of hepatic metastases, and it has the ability of identifying small parenchymatous lesions, and the size and grade of hepatic affection.^12^ The use of echography in the transoperative period can detect occult colorectal metastasis that was not visualized by CT, with 96% global sensitivity. It is also useful for demonstrating hepatic segmental anatomy, acquiring relevance when the tumor is in close proximity to the blood vessels. The value of the intraoperative echography is operator-dependent, but in expert hands, it has been demonstrated that it alters the surgical plan in 20% of patients.^3^

The diagnostic laparoscopy is useful prior to hepatic resection, aiding in the identification of lesions not observed during preoperative imaging. Carrying out the laparoscopy in terms of time, expenses, and morbidity has not demonstrated its performance as suggested in generalized practice. A high clinical risk score has been developed to clarify the performance of laparos-copy prior to the resection, including variables, such as the carcinoembryonic-antigen level, status of the primary-tumor lymphatic ganglion, the disease lapse (from diagnosis to diagnosis of the hepatic metastasis), and the number and size of the hepatic tumors. This preoperative index is helpful for staging patients with a high risk of earlier recurrence and can aid in determining patients who require neoadjuvant therapy. Also, it can determine disease extension prior to an aggressive surgical approach.^3^

### Cytology by Fine-needle Aspiration

This is a very well-established diagnostic method with the benefit of histopathologic confirmation of the diagnosis. There is evidence of the association of the biopsy with hemorrhage, pneumothorax, and extrahepatic dissemination of the tumor by cell implantation; this represents a reduction in the survival rate even if resection of the hepatic metastasis is carried out.^3,4,12^ The risk involved in the performance of this procedure should be sufficient to decrease the number of unjustified cases.

### Biomarkers

Molecular detection of colorectal cancer offers the advantage of its being a minimally invasive technique. Ideally, a biomarker should be able to determine the difference between cancer and advanced adenomas from other lesions, being released in a continuous way into the intestinal lumen or circulation, and disappearing or decreasing after the resection or treatment of the lesion. The stool sample is based on the fact that, at an early stage, cancer can bleed and release cells into the intestinal lumen. SEPT9 is a guanine triphosphatase; its hypermethylation of its promoter region being associated with colorectal cancer. Aberrant methylation of SEPT9 at the tissue level discriminates between a neoplasm and normal mucous. The methylation test has 50 to 70% sensitivity and 85 to 90% specificity.^1^ The availability of biomarkers that distinguish between treatment response and early recurrent cases after radiotherapy would represent important clinical progress for defining high-risk patients. Ceramide is a proapoptotic sphingolipid generated after radiation in the outer layer of the outer cellular-membrane layer by the hydrolysis of the sphingomyelin of sphingomyelinase acid or of the neutral sphingomyelinase; it is synthesized *de novo* in the endoplasmic reticulum. Dubios et al^40^ compared the levels of pre- and postoperative ceramide with resection of the tumor, observing that total levels of ceramide and of the four main subtypes were higher on days 3 and 10 of treatment, with an objective response. According to Kaplan-Meier curves, total control of the tumor was achieved in 1 year in patients with increasing total levels of ceramide, while 50% of patients with a decrease in these levels experience an increase in tumor volume.

High levels of the carcinoembryonic antigen (CEA) in the preoperative period predict unsuccessful results in resection of the hepatic metastasis, while high levels in the postoperative period comprise the first clue of local or distant recurrence in an asymptomatic patient.

However, an increasing concentration of the CEA can be a relatively delayed phenomenon in patients with hepatic metastasis. The CEA can be increased in 90% of patients with hepatic metastasis, and is useful for follow-up of patients with colorectal cancer and presenting a sensitivity and specificity of 75 and 90 to 95% respectively, in the detection of recurrence.^4^ In a review of 1,001 patients on whom a hepatic resection was performed due to colorectal metastasis, a level of CEA of >200 ng/mL was described as a negative predictive factor and presented with a mean survival of 24 months, while patients with preoperative levels <200 ng/mL had a mean survival of 38 months. Despite this clinical correlation, preoperative levels of CEA are not a reason to prevent a potential, curative hepatic resection.^41^

In patients in whom an apparently curative resection was performed, a follow-up must be established to detect metastatic disease, with the expectancy of performing early diagnosis and disease management that will end in better patient quality of life.^4^

## TREATMENT

The main objectives of management in colorectal cancer include ensuring a good quality of life with the highest survival rate possible and with current management of the surgical resection of the associated metastasis, which ensures a high life expectancy and low mortality.^3^ The cure is not a realistic goal in the majority of patients with hepatic metastasis; therefore, establishing early, specific treatment after a comprehensive analysis of the diagnosis, determination of tumor extension, and associated prognosis are suggested.

### Surgical Treatment

Resection of the metastasis is the only treatment that offers the possibility of cure and it has proven to contribute to patient survival.^42^ There remains controversy in terms of the extension necessary for the resection. Some authors recommend that the margin must be 1 cm or more.^43,44^ However, in other studies, there is no significant difference in long-term prognosis with margins of <1 cm, as long as it is an R0 resection.^45,46^ The procedure is very safe, with mortality <5%. Mean hospital stay was 5 to 7 days for hepatic resection and 7 to 10 days for any other type of major resection.^47^ In some types of cancer, such as pancreatic, the performance is taken into account of the hepatectomy associated with resection of the neighboring organ, considered a safer surgical procedure and offering higher survival rates. In patients with hepatic metastasis from breast cancer, the treatment-of-choice is surgical; however, in patients with a low prognosis, it is worthwhile to value the risk-benefit, due to which the risk is greater. Mean follow-up for patients who are surgically treated is approximately 40 months. Mean disease-free survival is from 32.2 months, and mean time until disease progression is 17.7 months.^48^ In the majority of hepatic metastases, surgical resection offers the sole therapeutic possibility. In association with hepatic and systemic arterial infusion, chemotherapy, mainly in tumors that cannot be resected, can be employed as adjuvant treatment after hepatic resection.^49^

In neuroendocrine tumors, hepatic metastasis is an indicator of poor prognosis; in this case, complete surgical resection is best therapeutic choice. There are other medical and surgical minimally invasive options, which include ablation techniques, such as the following: Radio-frequency, microwave therapy, cryotherapy, transcatheter embolization, chemoembolization, radioembolization, and chemotherapy with somotostatin or interferon analogs. There is no evidence, to our knowledge, that compares medical options and alternative surgical treatments. An aggressive surgical approach, in addition to procedures directed to the liver, is recommended to prolong the global survival rate.^50^

### Chemotherapy and Surgery

Despite the advances in chemotherapy, surgery remains the treatment-of-choice, surpassing other treatments, such as cryosurgery or radiofrequency ablation.^42^ During the last 30 years, the benefits have been established of surgical resection and systemic chemotherapy. In reality, surgical resections are more feasible, entertaining very low mortality and a 5-year survival of nearly 40%; however, only 10 to 20% of patients are candidates for surgery. The benefits of chemotherapy are currently being described. Tumor reduction after preoperative administration of chemotherapy and the availability of ablation techniques allows for a treatment with curative intentions in metastases initially considered as unresectable.^51^ Prognosis of hepatic metastases that are unresectable have been managed in recent years in association with chemotherapy and surgical resection as part of a multidis-ciplinary workup. The 5-year survival rate after hepatic resection is 25 to 40%. Synchronous or metachronous hepatic metastases that are resectable must be treated with preoperative chemotherapy during 3 months with FOLFOX4 (Oxaliplatin, Folinic acid, and 5-Fluorouracil). Chemotherapy must be administered before the surgical procedure and 3 months after surgery. In the case of primary surgery, adjuvant chemotherapy starts with 5-FU LV, FOLFOX4, XELOX, or FOLFIRI. In disease, i.e., potentially resectable, primary chemotherapy is based on a more intensive regimen, such as FOLFIRINOX, and must be considered to increase the chances of cure. Palliative chemotherapy is based on FOLFIRI or FOLFOX4/XELOX with or without focus therapy; this comprises the cornerstone of treatment for unresectable disease.^8,52^

A total of 24.5% of patients subjected to liver resection will present a recurrence exclusively at the hepatic level, and 20.8% will be subjected to a new resection.^53^ When there are multiple lesions in one hepatic lobe or when an infiltration occurs, segmental hepatectomy or hemi-hepatectomy is the treatment-of-choice. When the volume of the residual liver is inadequate, preoperative embolization of the portal vein must be considered. This is advisable in patients in whom the grade of the surgical resection results in a hepatic volume of <25 to 40%, which is less than having optimal liver function and preventing postoperative liver insufficiency.^54,55^

### Treatment of Unresectable Metastases

Isolated hepatic perfusion (IHP) is an optional regional treatment that offers a high dose of chemotherapy, biological agents, and hyperthermia by means of a recirculation circuit of vascular perfusion as treatment of hepatic metastasis. A study was conducted of IHP with tumor necrosis factor plus Melphalan, or IHP with Melphalan alone, Floxuridine in infusion, and Leucovorin in patients with advanced hepatic metas-tases from colorectal cancer that were unresectable or recurrent. It was concluded that IHP can be performed with low morbidity and that it possesses great antitumor activity with clinical relevance in patients with hepatic metastasis from colorectal cancer that are unresectable or recurrent.^56^ About 10 to 25% of patients with isolated metastases in the liver are candidates for resection due to anatomical limitations (localization or extension of the metastatic lesions), inadequate functional-liver reserve, or comorbidities. The hepatic metastases of colorectal cancer are defined as resectable when it is anticipated that these can be completely resected, when there is adequate vascular flow (entry and exit), preserved bile drainage, and adequate hepatic volume. For cases that are unresectable, local therapy is the best choice; due to that it increases the survival rate.^57^

## CONCLUSION

Colorectal cancer is a relevant disease worldwide, especially in Western countries and in developing countries, presenting high morbidly and mortality. Knowledge is vital on the disease’s predisposing factors, mechanisms, diagnostic methods, and the treatment of hepatic metastasis due to its anatomical situation in the abdominal cavity.

The adoption is highly significant of more and better programs in the health system, with main objectives with respect to prevention, early diagnosis, and adequate treatment, which will aid in the survival and prognosis of the patients.
